# Supplementary figures and images for: Initial-state-dependent, robust, transient neural dynamics encode conscious visual perception
Source: PLoS Comput Biol. 2017 Nov 27;13(11):e1005806. doi: 10.1371/journal.pcbi.1005806 (PMC5720802; doi:10.1371/journal.pcbi.1005806)

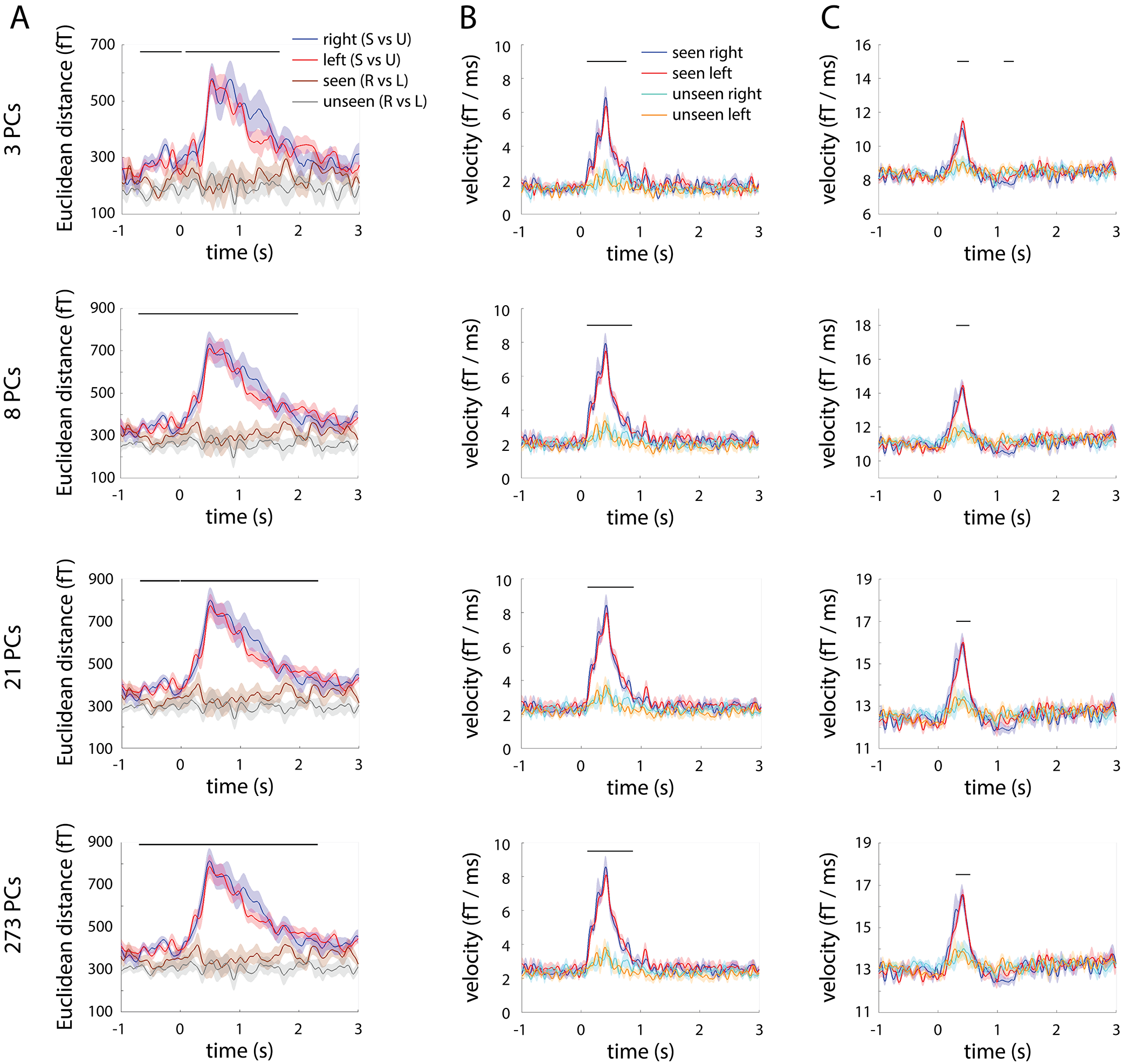

Supplement: S1 Fig — (A) Same as Fig 2D, but using 3, 8, 21, or 273 PCs. (B-C) Same as Fig 4A–4B, but using 3, 8, 21, or 273 PCs. (TIF) [file pcbi.1005806.s001.tif]

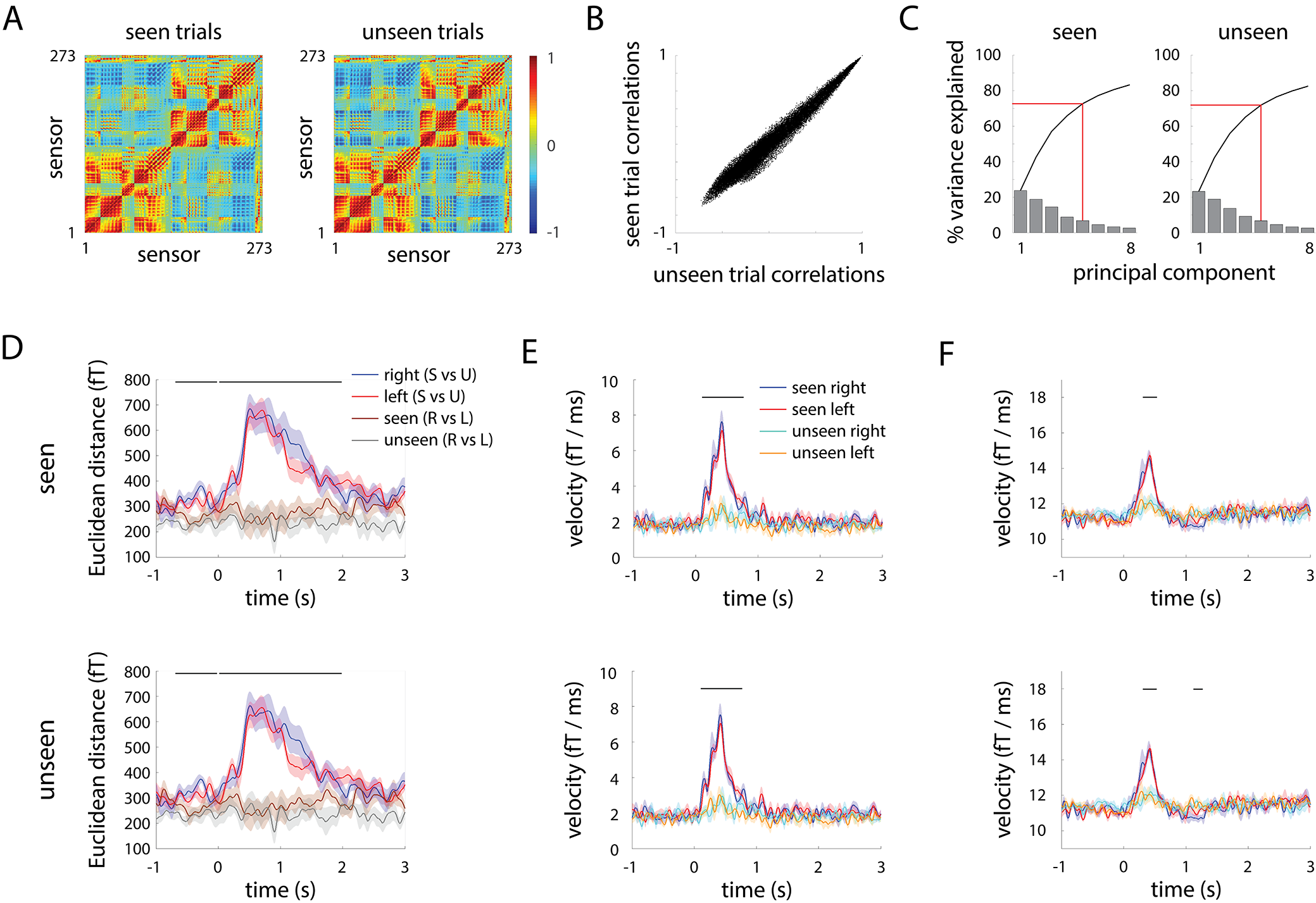

Supplement: S2 Fig — (A) Sensor-by-sensor correlation matrix computed using temporally concatenated seen (left) or unseen (right) trials from an example subject. These correlation matrices were used for PCA decomposition. (B) Element-by-element scatter plot of the correlation matrices in A. (C) Variance explained by each PC when PCA was conducted on seen trials (left) or unseen trials (right) alone. The top five PCs explain >70% of variance in both cases and are used for subsequent analyses. (D-F) Euclidean distance (D), trial-averaged trajectory velocity (E), and single-trial trajectory velocity (F). Top: PC coefficients extracted from seen trials alone are applied to both seen and unseen trials. Bottom: PC coefficients extracted from unseen trials alone are applied to both seen and unseen trials. All analyses use 0.05–5 Hz activity. (TIF) [file pcbi.1005806.s002.tif]

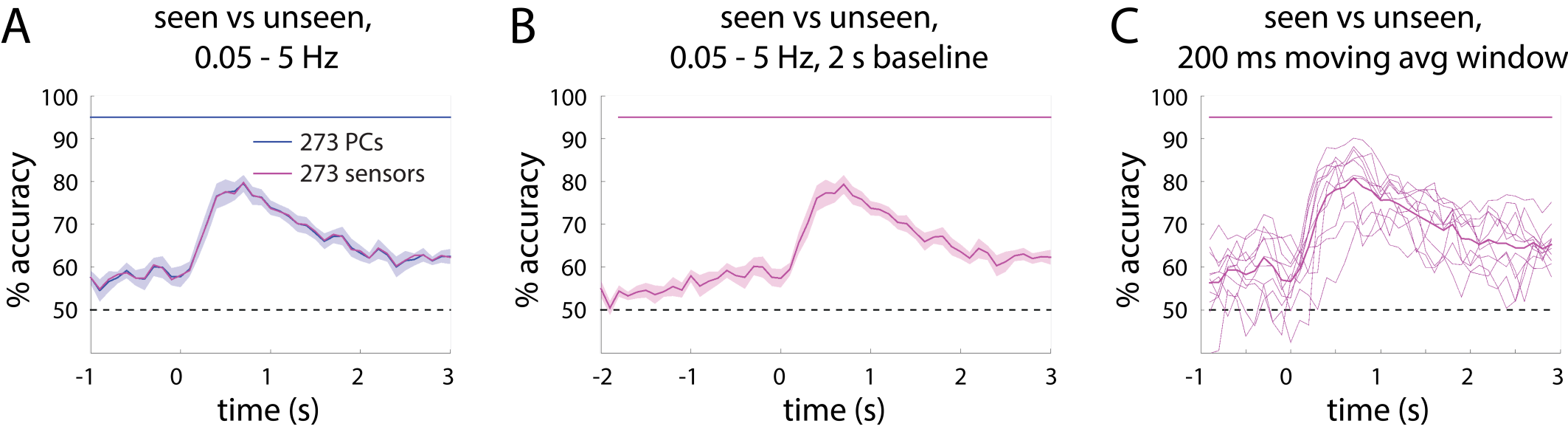

Supplement: S3 Fig — (A)The magenta trace is reproduced from Fig 5A, showing the accuracy of single-trial classification of seen vs. unseen perceptual outcome, using filtered SCP activity across 273 sensors. Blue trace plots seen vs. unseen decoding accuracy when the classifier was constructed using activity across 273 PCs; shaded area shows s.e.m. across subjects. The accuracy obtained using 273 PCs is significantly above chance at every time point from 1 sec before to 3 sec after stimulus onset (blue horizontal bar, p < 0.05, cluster-based permutation test). (B) Seen vs. unseen decoding in an extended pre-stimulus period (showing mean and s.e.m. across subjects). Decoding performance was significantly above chance up to 1.8 s prior to stimulus onset (horizontal bar, p < 0.05, cluster-based permutation test). (C) Seen vs. unseen decoding result using a moving-averaging window (200-ms-length, half-overlapping) applied to the full-band data. Thin traces are the decoding results from each of the 11 subjects; thick trace is the group average. Magenta horizontal bar shows that at the population level, the result is significant at every time point (p < 0.05, cluster-based permutation test). Single-subject statistics are reported in Results. (TIF) [file pcbi.1005806.s003.tif]

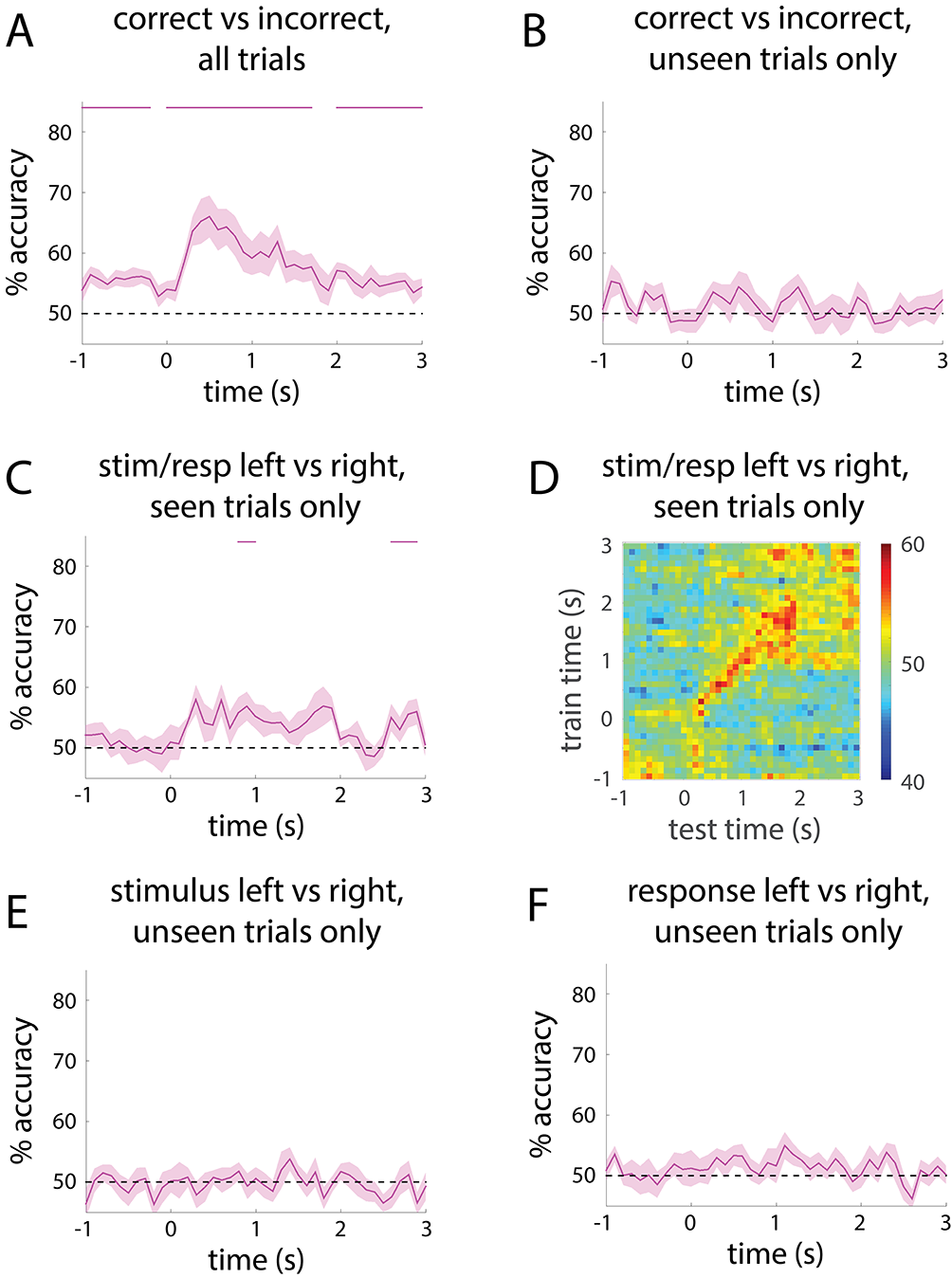

Supplement: S4 Fig — (A) Decoding of correct vs. incorrect orientation discrimination performance. (B) Decoding of correct vs. incorrect performance for unseen trials only. (C) Decoding of stimulus orientation / discrimination response (left vs. right) for seen trials only. Note that due to the removal of a very small number of seen & incorrect trials, seen trials were always correct; hence, stimulus orientation and discrimination response are identical for analyzed seen trials. (D) Temporal generalization of decoding result in C. Rows indicate time points used to train the SVM classifier and columns indicate time points used for testing. (E-F) Decoding of stimulus orientation (E) and discrimination response (F) for unseen trials only. Shaded areas represent s.e.m. across subjects and horizontal bars show time points where decoding accuracy is significantly above chance (p < 0.05, cluster-based permutation test). (TIF) [file pcbi.1005806.s004.tif]

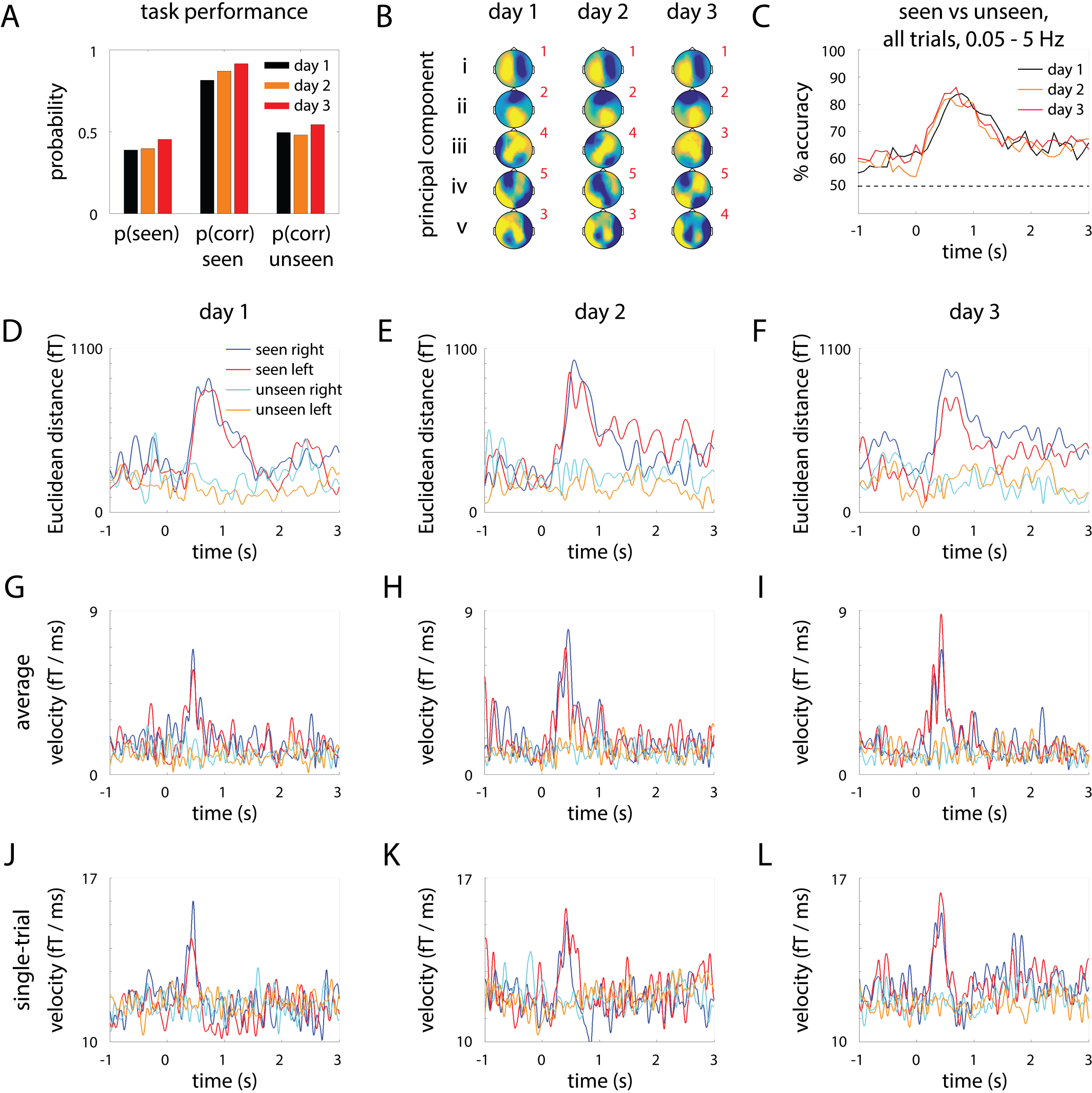

Supplement: S5 Fig — To assess the robustness and reliability of the main findings, two additional subjects (#12 & #13) performed the same task in three experimental sessions on three separate days. The main findings reported in the main subject group replicated in subject #12 and were consistent across sessions. (A) Task performance (cf. Fig 1B). (B) Aligned PC topographies (cf. Fig 3A). (C) Single-trial decoding of seen vs. unseen perceptual outcome (cf. Fig 5A, magenta trace). (D) Euclidean distance between experimental conditions in the state space defined by the top 5 PCs (cf. Fig 2D). (E) Trial-averaged trajectory velocities (cf. Fig 4A). (F) Single-trial trajectory velocities (cf. Fig 4B). B-F were calculated using SCP activity (0.05–5 Hz filtered activity). (TIF) [file pcbi.1005806.s005.tif]

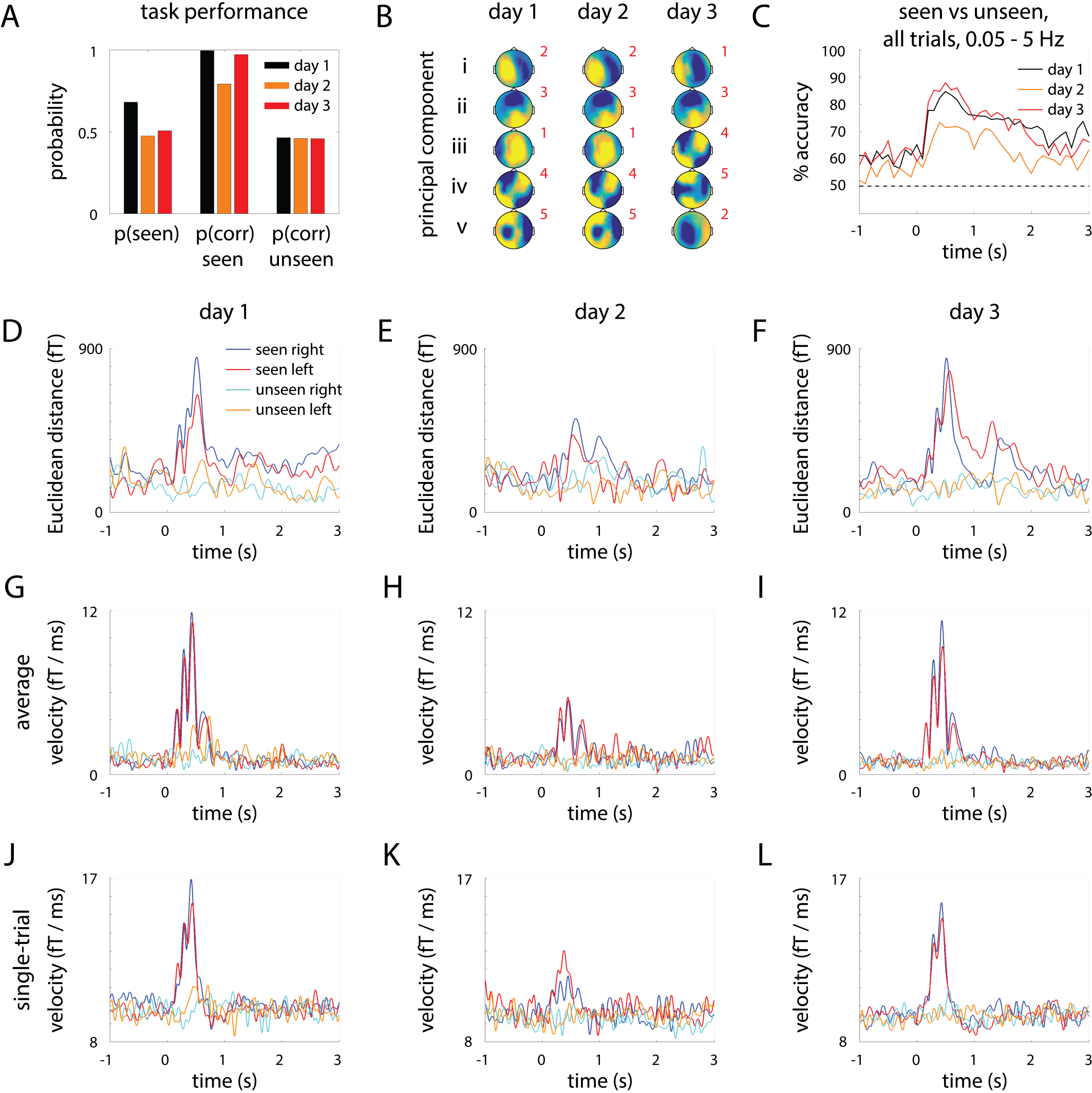

Supplement: S6 Fig — Same as S5 Fig, but for subject #13. The neural effects in subject #13’s data from day 2 were relatively low (C-F); in conjunction with the unusually low orientation discrimination accuracy in seen trials on day 2 (A), this may suggest that the subject simply performed abnormally poorly on this day, perhaps due to distraction or lack of sleep. Nonetheless, the direction of the effects on day 2 was consistent with days 1 and 3. (TIF) [file pcbi.1005806.s006.tif]
